# Exploring *Campylobacter* seasonality across Europe using The European Surveillance System (TESSy), 2008 to 2016

**DOI:** 10.2807/1560-7917.ES.2019.24.13.180028

**Published:** 2019-03-28

**Authors:** IR Lake, FJ Colón-González, J Takkinen, M Rossi, B Sudre, J Gomes Dias, L Tavoschi, A Joshi, JC Semenza, G Nichols

**Affiliations:** 1 School of Environmental Sciences, UEA, Norwich, United Kingdom; 2 European Centre for Disease Prevention and Control, Stockholm, Sweden; 3 Centre for Radiation, Chemical and Environmental Hazards, Public Health England, London, United Kingdom; 4 Centre for Infections, Public Health England, London, United Kingdom; 5 University of Exeter, Exeter, United Kingdom

**Keywords:** *Campylobacter*, campylobacteriosis, climate change, food-borne infections, gastrointestinal disease, laboratory surveillance, surveillance

## Abstract

**Background:**

Campylobacteriosis is the most commonly reported food-borne infection in the European Union, with an annual number of cases estimated at around 9 million. In many countries, campylobacteriosis has a striking seasonal peak during early/mid-summer. In the early 2000s, several publications reported on campylobacteriosis seasonality across Europe and associations with temperature and precipitation. Subsequently, many European countries have introduced new measures against this food-borne disease.

**Aim:**

To examine how the seasonality of campylobacteriosis varied across Europe from 2008–16, to explore associations with temperature and precipitation, and to compare these results with previous studies. We also sought to assess the utility of the European Surveillance System TESSy for cross-European seasonal analysis of campylobacteriosis.

**Methods:**

Ward’s Minimum Variance Clustering was used to group countries with similar seasonal patterns of campylobacteriosis. A two-stage multivariate meta-analysis methodology was used to explore associations with temperature and precipitation.

**Results:**

Nordic countries had a pronounced seasonal campylobacteriosis peak in mid- to late summer (weeks 29–32), while most other European countries had a less pronounced peak earlier in the year. The United Kingdom, Ireland, Hungary and Slovakia had a slightly earlier peak (week 24). Campylobacteriosis cases were positively associated with temperature and, to a lesser degree, precipitation.

**Conclusion:**

Across Europe, the strength and timing of campylobacteriosis peaks have remained similar to those observed previously. In addition, TESSy is a useful resource for cross-European seasonal analysis of infectious diseases such as campylobacteriosis, but its utility depends upon each country’s reporting infrastructure.

## Introduction

Campylobacteriosis, infection with *Campylobacter*, is the most commonly reported food-borne illness in the European Union (EU). It is reported to cause over 200,000 human cases annually, but—due to under-reporting—the actual number of infections may be closer to 9 million [1]. While most infections are self-limiting, campylobacteriosis has also been associated with Guillain-Barré syndrome (a temporary, rapid-onset paralysis that can result in permanent weakness and death), irritable and inflammatory bowel syndrome and, occasionally, reactive arthritis [2]. In the EU, the associated annual cost in terms of public health and lost productivity is estimated at EUR 2.4 billion [3].

A striking characteristic of campylobacteriosis epidemiology is the strong seasonal peak of cases observed in many temperate countries during early to mid-summer [4,5]. However, the underlying determinants of this seasonality are largely unknown. A number of studies have been conducted to identify factors that may contribute to this seasonal peak. These include changing bacterial colonisation patterns in broiler flocks, changes to food preparation practices (e.g. the increased use of barbeques in summer), transmission through flies and seasonal changes in recreational activities [6,7]. Epidemiological studies have demonstrated associations between campylobacteriosis cases and both temperature and precipitation [8,9], although the mechanisms for these remain elusive. Exposure through food-borne routes are associated with around 50% of *Campylobacter* cases [10].

In the early 2000s, two publications examined *Campylobacter* seasonality focusing on cross-national differences in seasonal patterns, as well as associations with weather variables [5,11]. One of these studies also explored associations with weather (temperature and precipitation) [11]. Both studies included data from several European and non-European countries that differ in their reporting systems. Since then, a number of new interventions have been implemented in several European countries to control food-borne illness, including campylobacteriosis [1]. It is therefore warranted to re-examine the seasonal patterns of campylobacteriosis, as well as associations with weather variables, by using data from across Europe that was obtained through more consistent reporting systems. One challenge in previous cross-national campylobacteriosis studies was obtaining comparable incidence data. Previous work [5,11] overcame this issue by contacting numerous individual experts at national surveillance centres.

Within Europe, a potential new solution to obtain comparable incidence data between countries emerged when the European Centre for Disease Prevention and Control (ECDC) was established in 2005, with a mandate to collect, examine and disseminate surveillance data on over 50 infectious diseases from EU and European Economic Area (EEA) countries. Data collection for campylobacteriosis started in 2008 for 2007 data, and continues to occur annually. The submission and validation of data are the responsibility of the European networks of national disease surveillance experts, which are coordinated by ECDC. The platform for the submission, validation, storage and dissemination of these data is the European Surveillance System (TESSy) [12]. The validated data are published in the Surveillance Atlas of Infectious Diseases on the ECDC website [13]. While the Surveillance Atlas provides a good epidemiological overview of infectious diseases in the EU/EEA, it only allows analyses by year. However, the collected data in TESSy allows for more detailed analyses by shorter time periods, thereby enabling connections to environmental parameters. To access the aggregated TESSy data, such as that presented in this paper, researchers must submit a CV, an outline of proposed research and its likely outputs to ECDC for approval [12].

The aims of this work are: (i) to examine how the seasonality of campylobacteriosis infections varies across Europe and to explore associations between incidence, temperature and precipitation and (ii) to compare these results to previous studies. We also sought to assess the utility of data from TESSy for cross-European seasonal analysis of campylobacteriosis.

## Methods

### Campylobacteriosis data

For all EU/EEA countries reporting campylobacteriosis cases (27 of the 30 EU/EEA countries), a country-level weekly number of confirmed campylobacteriosis cases occurring between January 2008–December 2016 was obtained from TESSy. Data before 2008 were excluded, as they have not been validated. In TESSy, campylobacteriosis cases are reported according to the EU case definition at the time of reporting [14,15]; however, there were no changes in the definition during the study period [13].

Within TESSy, countries can report six types of dates: date of symptom onset, date of diagnosis, date of receipt of the sample at the source laboratory, date of receipt of the sample at the reference laboratory, date of notification and date used for statistics. The latter is mandatory and countries may decide which date to use for statistical purposes; most countries report the date of notification for statistics, which is what was used in this study to determine the week for analyses. For January 2008–December 2016, annual country populations were obtained from the World Bank [16] and were interpolated to a weekly level; campylobacteriosis case counts were then divided by the population size to provide a weekly crude incidence rate (cases/100,000 population) for each week. For the purpose of this study, our interest is in indigenous (autochthonous) cases, hence cases reporting foreign travel (n = 135,178; 6.83%) were removed from the dataset and analysis. Table 1 presents the total, mean annual and mean weekly number of confirmed campylobacteriosis cases and the reported incidence by country. There were over 1.8 million confirmed campylobacteriosis cases reported from the 27 EU/EEA countries.

**Table 1:** Total, mean annual and mean weekly confirmed reported campylobacteriosis cases by country, EU/EEA^a^, 2008–2016 (n = 1,844,004)

Countries	Campylobacteriosis cases										Mean annual count	Mean annual incidence (cases/100,000 population)	Mean weekly count	Mean weekly incidence (cases/100,000 population)
	2008	2009	2010	2011	2012	2013	2014	2015	2016	Total				
Austria	3,857	4,150	3,968	5,073	4,695	5,159	5,922	5,645	6,460	44,929	4,992	59.0	95.8	1.1
Belgium	5,079	5,684	6,038	7,669	6,771	8,117	7,963	NA	NA	47,321	5,258	47.9	129.6	1.2
Cyprus	23	37	55	62	68	57	39	29	21	391	43	3.9	1.7	0.1
Czech Republic	19,847	20,035	20,869	18,479	18,102	17,971	20,501	20,670	23,811	180,285	20,032	191.1	384.4	3.7
Denmark	2,898	2,903	3,378	3,392	3,199	3,193	3,218	3,670	3,824	29,675	3,297	59.0	63.3	1.1
Estonia	132	159	178	195	258	360	268	300	281	2,131	237	17.9	4.9	0.4
Finland	1,963	1,582	1,536	1,866	2,289	2,043	2,415	2,702	2,788	19,184	2,132	39.4	40.9	0.8
France	3,300	3,832	4,211	5,383	5,086	5,242	5,934	6,065	6,676	45,729	5,081	7.7	97.5	0.1
Germany	60,522	58,764	61,104	66,214	58,278	58,165	64,798	63,792	68,086	559,723	62,191	76.4	1,193.4	1.5
Hungary	5,489	6,582	7,157	6,102	6,413	7,238	8,406	8,352	8,513	64,252	7,139	71.9	137.0	1.4
Iceland	93	46	33	78	30	61	83	61	70	555	62	19.1	2.3	0.7
Ireland	1,728	1,770	1,614	2,426	2,394	2,284	2,577	2,438	2,503	19,734	2,193	47.7	42.1	0.9
Italy	260	505	397	441	727	1,138	1,186	962	1,007	6,623	736	1.2	14.9	0.0
Latvia	NA	NA	NA	7	8	9	37	74	89	224	37	1.3	1.9	0.1
Lithuania	759	817	1,089	1,124	926	1,126	1,170	1,178	1,217	9,406	1,045	34.6	20.1	0.7
Luxembourg	464	521	600	704	587	676	866	254	518	5,190	577	109.8	11.1	2.1
Malta	77	130	203	216	223	243	288	248	210	1,838	204	48.0	4.6	1.1
Netherlands	3,179	3,562	4,071	4140	4,077	3,505	3,976	3,562	3,071	33,143	3,682	22.0	70.8	0.4
Norway	1,329	1,498	1,305	1,524	1,523	1,539	1,832	1,131	1,201	12,882	1,431	28.8	27.5	0.6
Poland	267	357	365	352	429	551	643	650	769	4,383	487	1.3	20.3	0.1
Portugal	NA	NA	NA	NA	NA	NA	1	266	356	623	208	0.7	6.0	0.1
Romania	NA	NA	NA	149	92	217	256	311	517	1,542	257	0.9	5.8	0.0
Slovakia	3,048	3,784	4,457	4,541	5,686	5,819	6,705	6,903	7,581	48,524	5,391	99.7	103.5	1.9
Slovenia	898	948	1,017	991	998	1,003	1,179	1,294	1,622	9,950	1,106	53.9	21.2	1.0
Spain	5,414	5,279	6,304	5,473	5,777	7,436	11,818	13,541	15,848	76,890	8,573	18.5	164.5	0.4
Sweden	2,279	7,167	3,531	3,659	3,504	3,708	4,139	5,051	7,156	40,194	4,466	46.9	85.7	0.9
UK	54,570	64,089	69,613	71,530	72,463	65,544	66,053	57,687	57,134	578,683	64,298	101.4	1,233.9	1.9
Total	177,475	194,201	203,093	211,790	204,603	202,404	222,273	206,836	221,329	1,844,004	204919	41.9	3927.3	0.8

EU/EEA: European Union/European Economic Area; UK: United Kingdom. NA: not applicable.

^a^ Twenty-seven of 31 countries reporting.

**Figure 1:**
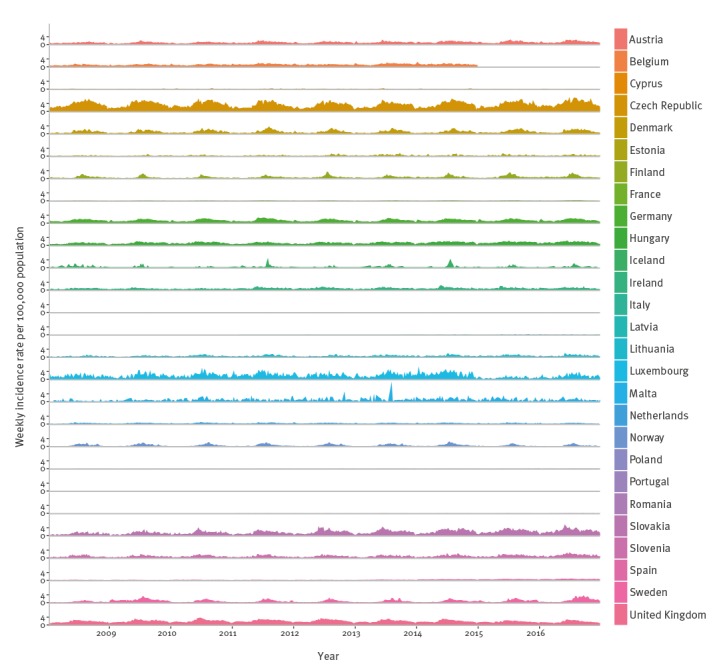
Weekly confirmed reported campylobacteriosis cases by country, EU/EEA^a^, 2008–2016 (n = 1,844,004)

The time series of the weekly crude campylobacteriosis incidence is presented in Figure 1. This indicates large differences in reported incidence between countries (e.g. Lithuania vs Luxembourg), as well as variability in the seasonal patterns between different countries (e.g. Norway vs Germany).

EU/EEA: European Union/European Economic Area.

^a^ Cases per 100,000 population.

^b^ Twenty-seven of 31 countries reporting.

### Meteorological data

Average country-wide meteorological data (January 2008–December 2016) were linked to the weekly country-specific epidemiological data. These were sourced from high-resolution gridded datasets of daily mean air temperature (°C) and total precipitation (mm/day ^− 1^) for the whole of Europe, obtained from the E-OBS dataset from the EU-Framework Programme 6 project ENSEMBLES [17]. Temperature data were retrieved at a 0.25 × 0.25 degree resolution and precipitation at a 0.5 × 0.5 degree resolution for land cells only. Weekly mean temperature and weekly total precipitation were computed using standard methods in the Climate Data Operators software (Max Planck Institute, Hamburg, Germany) [18], and country-wide mean weekly temperature and mean weekly precipitation were calculated using standard routines within the ‘raster’ package for R Version 2.4-15 (R Development Core Team) [19,20].

### Strength and timing of seasonal peak

The first stage of the analysis was to estimate the strength and timing of the seasonal campylobacteriosis peak by country. To perform this analysis, we excluded countries with more than 10% of weeks missing campylobacteriosis data or a mean weekly count of less than 10 campylobacteriosis cases, since below this threshold it would be difficult to identify a robust seasonality. This exclusion criteria removed Belgium, Cyprus, Estonia, Iceland, Latvia, Malta, Poland, Portugal and Romania. The analysis progressed with data for 1,784,996 campylobacteriosis cases from 18 countries.

We smoothed the time series of the 18 countries using methods adopted in Nylen et al. (kernel smoothing) [5] to control for some of the random fluctuations in incidence and to allow for a direct comparison between studies. From this smoothed series, the average weekly cycle was calculated, pooling individual year’s data. The week of maximum incidence was subsequently extracted.

To estimate the size of the seasonal peak, the proportions of the annual cases occurring during the peak week and for one week on either side were calculated (i.e. a 3-week period). The proportion of cases occurring during the peak week and 2, 3 and 4 weeks on either side were also calculated, in line with the study by Nylen et al [5]. In addition, so that the timing of the peak week count could be compared with results from another publication by Kovats et al. [11], the peak week was calculated using the raw data and averaged between years.

### Seasonal clustering

In order to explore the clustering of seasonal patterns for campylobacteriosis cases across Europe, we analysed weekly campylobacteriosis incidence for the 18 countries for which a robust seasonality could be identified. The kernel-smoothed, country-specific campylobacteriosis annual cycles were normalised (with zero mean and unit variance) to adjust for the different absolute levels of incidence between countries. These data were also detrended using a linear function of time to remove long-term trends in reported incidence, because it is impossible to ascertain whether these represent actual changes in incidence or merely changes in reporting [21].

Using these data, the analysis clustered countries into groups in order to identify those with similar seasonal patterns using Ward’s Minimum Variance Clustering (WMVC) method. This method is based on the linear model sum of squares criteria to produce groups that minimise the within-group sum of squares. We selected WMVC because it is less susceptible to noise and outliers than other methods, such as k-means [22]. The output of the clustering algorithm is a dendrogram, indicating the similarity of mean weekly seasonal trends between countries. This dendrogram is cut to produce clusters. Membership to a cluster was binary and exclusive, such that a country could only belong to one cluster after the partition. Cluster analysis such as this is a data exploration technique and there is no set value defining where the dendrogram should be cut. We emphasise that the clustering algorithm identifies similarities in the mean weekly seasonal trends of *Campylobacter* incidence and not in the absolute number of cases. The analysis was repeated using the raw unsmoothed data, but the same clusters were produced. WMVC was performed using the R ‘stats’ package.

### Indigenous campylobacteriosis incidence and associations with temperature and precipitation

To explore the relationships between indigenous campylobacteriosis incidence and meteorological variables, the second stage of the analysis investigated the short-term effects of temperature and precipitation upon weekly campylobacteriosis incidence. As these relationships may be lagged and nonlinear, they were estimated using a two-stage multivariate meta-analysis methodology [23]. In the first stage, generalised linear models with a quasi-maximum likelihood Poisson specification were used to account for possible over-dispersion in the campylobacteriosis incidence data [24]. Regression models were fitted for each country to derive country-specific estimates of the nonlinear and delayed association between campylobacteriosis and each predictor. In this stage, the estimated relations were entirely defined by the predictors. In the second stage, regression coefficients and confidence intervals (CI) were used in a multivariate, meta-analytical model to determine the mean relations across countries.

All statistical analyses were conducted in R Version 2.4-15.

## Results

**Figure 2:**
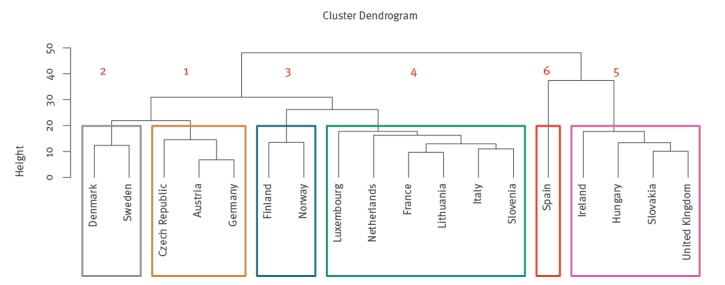
Dendrogram showing clusters of countries with seasonal trends in reported campylobacteriosis cases’ weekly incidence, 18 EU/EEA countries, 2008–2016

The analysis included a total of 1,784,996 cases from 18 countries for the period 2008–16. Table 2 provides the estimated week of peak campylobacteriosis cases for each country and the proportion of cases occurring near this estimated peak. It also indicates the cluster to which each country was assigned using hierarchical cluster analysis. The cluster dendrogram is provided in Figure 2, and was cut to produce six well-defined clusters.

**Table 2:** Week of maximum number of reported campylobacteriosis cases and proportion of cases occurring within a specified series of weeks during the estimated peak, EU/EEA countries^a^, 2008–2016 (n = 1,784,996)

Country	Nylen methodology [5] with this data					Results from Nylen study [5]		Kovats methodology [11] with this data	Results from Kovats study [11]
	Mean peak week	Proportion of cases within a number of weeks from peak				Mean peak week^b^	Proportion of cases + / − 4 weeks from peak	Mean peak week	Mean peak week^c^
		+ / − 1 week	+ / − 2 week	+ / − 3 week	+ / − 4 week				
Austria	27	0.09	0.14	0.19	0.25	NA	0.26	27	NA
Czech Republic	31	0.09	0.14	0.19	0.46	NA	NA	32	33
Germany	28	0.09	0.15	0.20	0.26	NA	NA	28	NA
**Cluster 1^d^ total**	**31**	**0.09**	**0.14**	**0.20**	**0.26**	NA	NA	**32**	NA
Denmark	32	0.13	0.21	0.28	0.34	32	0.30	32	32
Sweden	31	0.11	0.18	0.24	0.29	33	0.28	31	NA
**Cluster 2^d^ total**	**32**	**0.13**	**0.20**	0.27	**0.34**	NA	NA	**31**	NA
Finland	29	0.16	0.25	0.33	0.40	31	0.34	29	NA
Norway	30	0.16	0.26	0.35	0.42	NA	NA	30	NA
**Cluster 3^d^ total**	**29**	**0.16**	**0.25**	**0.33**	**0.40**	NA	NA	**30**	NA
France	34	0.09	0.15	0.20	0.25	NA	NA	29	NA
Italy	32	0.10	0.15	0.20	0.25	NA	NA	31	NA
Lithuania	31	0.09	0.15	0.21	0.26	NA	NA	31	NA
Luxembourg	25	0.09	0.14	0.19	0.24	NA	NA	24	NA
Netherlands	24	0.09	0.14	0.19	0.24	NA	NA	23	34
Slovenia	30	0.09	0.14	0.20	0.26	NA	NA	33	NA
**Cluster 4^d^ total**	**26**	**0.09**	**0.14**	**0.20**	**0.25**	NA	NA	**31**	NA
Hungary	23	0.08	0.13	0.17	0.22	NA	NA	24	NA
Ireland	21	0.09	0.14	0.18	0.23	NA	NA	20	31
Slovakia	24	0.10	0.16	0.22	0.27	NA	NA	23	NA
UK^e^	25	0.08	0.14	0.19	0.24	25	0.24	24	23
**Cluster 5^d^ total**	**24**	**0.07**	**0.12**	**0.16**	**0.21**	NA	NA	**23**	NA
Spain	22	0.07	0.12	0.16	0.21	NA	NA	23	26
**Cluster 6^d^ total**	22	0.07	0.12	0.16	0.21	NA	NA	23	26

EU/EEA: European Union/European Economic Area; NA: not applicable; UK: United Kingdom.

^a^ Eighteen countries.

^b^ Comparative data from [5]. Date is laboratory report date.

^c^ Comparative data from [11]. Date is calculated as two weeks post estimated date of onset.

^d^ Based upon all data within the cluster; hence, not the mean of the countries within each cluster.

^e^UK data from [5] are for Wales and Scotland only and do not include England or Northern Ireland; UK data from [11] are for England and Wales and do not include Scotland and Northern Ireland.

EU/EEA: European Union/European Economic Area.

**Figure 3:**
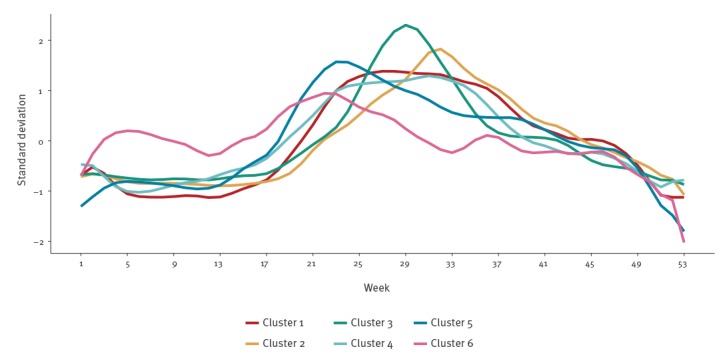
Seasonal trends in reported campylobacteriosis weekly incidence for the six clusters, 18 EU/EEA countries, 2008–2016 (n = 1,784,996)

Figure 3 plots the mean standardised seasonality for each of the six clusters. It is worth noting that although the cluster analysis groups similar countries into clusters, there is still a degree of similarity between the clusters, such as general increases in cases over the summer.

EU/EEA: European Union/European Economic Area.

Cluster 1: Austria, Czech Republic and Germany. Cluster 2: Denmark and Sweden. Cluster 3: Finland and Norway. Cluster 4: France, Italy, Lithuania, Luxembourg, the Netherlands and Slovenia. Cluster 5: Hungary, Ireland, Slovakia and United Kingdom. Cluster 6: Spain.

Here we present the results produced using the Nylen methodology; however, Table 2 indicates that the Kovats methodology produces similar results. Table 2 and Figure 3 indicate the campylobacteriosis seasonality and week of peak incidence for the 18 EU/EEA countries. Austria, Czech Republic and Germany are grouped within cluster 1, with a diffuse seasonal peak that occurs in week 31, but also a smaller peak around week 25 (Figure 3). The Nordic countries of Denmark and Sweden (Cluster 2), and Norway and Finland (Cluster 3), demonstrate a peak in weeks 32 and 29, respectively (Table 2). However, in these countries the peak is noticeably more pronounced than in cluster 1, with 34% (Cluster 2) and 40% (Cluster 3) of cases occurring within 4 weeks of the peak, vs 26% (Cluster 1); Norway has the highest number of cases occurring within 4 weeks of the peak, at 42%. Most other European countries are in cluster 4 (France, Italy, Lithuania, Luxembourg, the Netherlands, Slovenia), with a seasonal peak earlier in the year around week 26. Cluster 5 groups the United Kingdom (UK) and Ireland with the geographically separate countries of Hungary and Slovakia; peak incidence occurs in week 24, earlier than in clusters 1 to 4. This peak is more diffuse than in most other clusters, with only 21% of cases occurring within 4 weeks of the peak, and it is relatively asymmetric, with a higher rate of increase during early summer. Cluster 6 (Spain) is most dissimilar from the other clusters, with campylobacteriosis cases’ incidence more constant throughout the year and peak incidence less distinct than for most other clusters.

Table 2 also includes the peak week and the proportion of cases occurring within 4 weeks of this peak from two previous studies [5,11] using identical methodologies.

**Figure 4:**
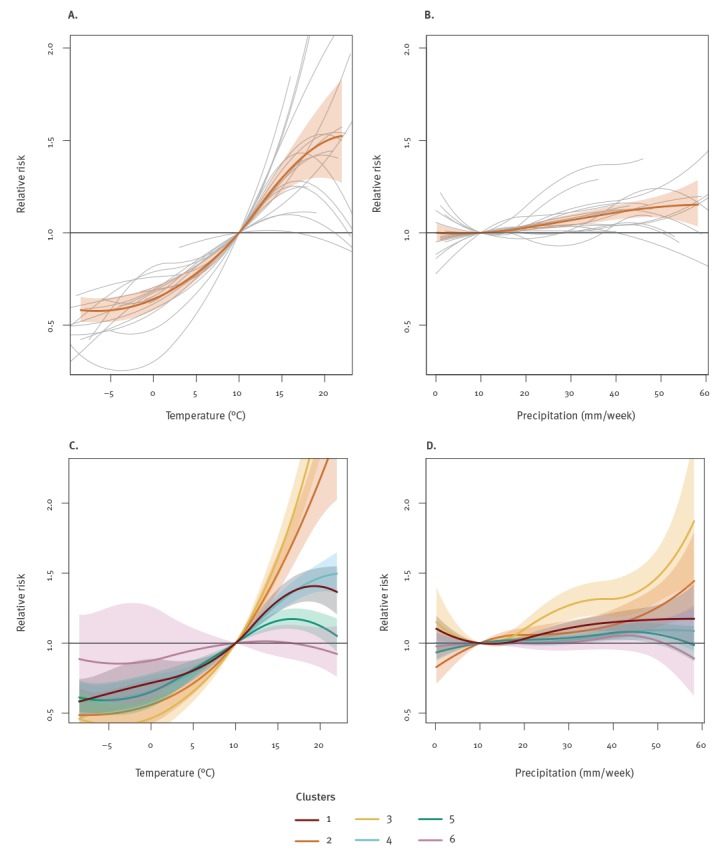
Influence of (A) temperature and (B) precipitation on the relative risk of *Campylobacter*¸ 18 EU/EEA countries, 2008–2016

Figure 4 depicts the estimated mean relationships between weekly campylobacteriosis incidence, temperature and precipitation across all 18 EU/EEA countries. These are interpreted as the accumulated effect of temperature or precipitation on incidence in the current week and up to 2 weeks later. The data show that as temperature increases so does incidence, although this effect is quadratic. Stratification by individual clusters indicates that, with the exception of cluster 6 (Spain), the 95% CI indicate a statistically significant positive association with temperature in all clusters. However, there is much variation between clusters and this positive association with temperature is greatest in clusters 2 and 3 (Nordic countries).

CI: confidence interval; EU/EEA: European Union/European Economic Area.

The thick lines indicate the maximum likelihood estimates and the shaded areas represent the 95% CI. The individual lines represent the mean relationship for each country (CI not shown to improve clarity).

Precipitation shows a quadratic-like effect on incidence, with the relative risk (RR) gradually increasing to 1.3 (95% CI: 1.1–1.5) until a precipitation of around 60 mm. The range of RR is lower for precipitation than for temperature, indicating that precipitation has less influence on campylobacteriosis incidence than temperature. Within the individual clusters, there are few associations with precipitation that are statistically significant (i.e. the 95% CI for the RR always includes 1). However, clusters 2 and 3 (Nordic countries) are an exception, as in this region there is a positive significant association with precipitation.

## Discussion

This article updates the results from two historical studies on campylobacteriosis seasonality and associations with temperature and precipitation. Using up-to-date data, cluster analysis was performed to group countries with similar seasonal patterns together. Furthermore, a contemporary two-stage multivariate meta-analysis methodology was used to explore associations with temperature and precipitation and to identify how these vary between clusters. We also took advantage of ECDC’s TESSy data on weekly national campylobacteriosis incidence. This permitted the inclusion of more European countries (18) than the previous research and allowed for an analysis of nearly 1.8 million indigenous campylobacteriosis cases, as per case definition.

Through this analysis, six clusters of countries were produced, indicating that within these clusters seasonality is comparable and, therefore, factors contributing to overall campylobacteriosis seasonality may also be similar. The countries in each cluster are geographically close to each other, with the exception of cluster 5. Within cluster 5, the UK and Ireland share land borders, as do Hungary and Slovakia, but these two pairs of countries are geographically separate. The reason for their clustering is unclear.

All the country clusters have a seasonal peak in incidence in early to mid-summer, corroborating past research [5,11]. The strongest seasonal peak of incidence was in Nordic countries (Clusters 2 and 3), confirming previous research [5]. In cluster 5 (Hungary, Ireland, Slovakia and the UK), incidence peaked around 6 weeks earlier than in more northern European countries (e.g. the Nordic countries in clusters 2 and 3). Past research has indicated that peak incidence in the UK occurs earlier than in other parts of Europe [11], but similar seasonality for Hungary and Slovakia has not been reported thus far. Ireland is also in this cluster, which contradicts past research suggesting a later peak in this country [11]. However, the earlier analysis was based on a low number of cases, potentially explaining this difference. In our analysis, Spain (cluster 6) had a more diffuse seasonal peak than other countries, which is in line with previous research [11] and could be a consequence of its southern location, low level of reported cases or small numbers of cases. The peak week within each cluster provides evidence of positive associations between the peak week and latitude, which may be related to the onset of spring and summer, as these seasons tend to occur progressively later with increasing latitude [25].

One observation in previous publications [5] is the consistency of the campylobacteriosis peak from year to year. Here we compare the peak dates and the campylobacteriosis incidence to data reported 15 years ago. Our estimate of the peak weeks and the proportion of cases occurring within 4 weeks of them were produced using identical methodologies to those in the two previous studies [5,11]. In terms of comparing the peak weeks between previous studies and this study, uncertainties in the data reported to TESSy and interannual variability indicate that small changes in peak weeks are unlikely to be notable.

A comparison of the peak weeks of campylobacteriosis incidence for those countries where a comparison is possible indicates little change in the peak week. Ireland is an exception, and we have already suggested that this may be due to small numbers of cases in the previous studies. The other exception where the peak week is different between this and other studies is the Netherlands, where the peak appears to have shifted earlier by several weeks; reported campylobacteriosis incidence in the country appears high in this and the previous paper [11], making the reason for this apparent change unclear. Our results indicate a peak week for the Netherlands that is similar to that for other neighbouring countries. For those countries where the shape of the campylobacteriosis curve is compared, we also show that the proportion of cases falling within 4 weeks of the peak appears to have remained similar. These two findings indicate that, in spite of numerous interventions to control campylobacteriosis [1], the shape and peak week of campylobacteriosis incidence has remained broadly similar. This similarity to previous years may also support the important role of the environment and weather conditions, which are minimally affected by food-based interventions. Indeed, a recent Danish study looking at IgG, IgA and IgM antibodies in the population suggested that exposure to *Campylobacter* has remained stable over a decade in spite of an increase in the reported number of cases [26].

A common feature of many ecological studies is the positive association between the incidence of campylobacteriosis cases and temperature [8,11]. It is therefore unsurprising that five of the six country clusters demonstrate significant associations with temperature. This 2-week lag fits with our understanding of the likely time between exposure, symptoms and the reporting of cases [11]. The seasonal patterns of campylobacteriosis and positive associations with temperature are poorly understood. *Campylobacter* survives better at low temperatures [27] and rarely replicates outside the mammalian gut. This complexity is observed in our data, as in clusters 2 and 3 campylobacteriosis incidence peaks before ambient temperature. In clusters 5 and 6, campylobacteriosis cases peak after peak ambient temperature. Therefore, associations with temperature are likely to be indirect, and proposed hypotheses have included changes in food preparation (e.g. using barbecues) [21], changes in visits to outdoor environments in which *Campylobacter* might be present (including outdoor swimming) [7,28], elevated consumption of fruit and salad (increasing the risk of cross-contamination) [21] or interactions between several of these factors [29]. Further studies have suggested the importance of environmental protozoa acting as an environmental reservoir [30].

In our analysis there was also an overall statistically significant association with precipitation. *Campylobacter* survival is enhanced in wet conditions [31] and the transmission of *Campylobacter* from the environment to humans may be greater during wetter conditions [7,32]. Some studies have also reported associations between campylobacteriosis and precipitation [9,33]. However, in our analysis the RR is small, and for most clusters the effect estimate is close to one. This may help to explain why the limited number of ecological studies examining the influence of precipitation found that it had no significant effect on campylobacteriosis incidence [4]. One interesting finding is that the associations between temperature, precipitation and *Campylobacter* infections are stronger in Nordic countries than in other European countries.

This research has a number of public health implications. The first is that knowledge about associations between disease and weather, in this case temperature and precipitation, can help in the development of early warning systems. It can also contribute to our understanding of the possible impact of climate change on incidence. However, because the relationships uncovered in studies such as this are associations, and the reasons behind these are not fully understood, they do not pinpoint specific interventions that should be taken during times when weather conditions are likely to elevate risk. For instance, during periods of elevated temperature cases of campylobacteriosis rise, but it is unclear whether this is due to, for example, changing food preparation methods (e.g. barbecues [22]) or visits to outdoor environments [7]. Given that there is uncertainty, it is difficult to ascertain the appropriate interventions.

The other public health benefit of this research is its highlighting of TESSy data as an important resource for the investigation of infectious disease occurrence in general across the EU/EEA. In this study, it was possible to obtain weekly time-series health data from across Europe from one comprehensive and standardised dataset available at EU/EEA level. Furthermore, standard EU case definitions aim to harmonise the categorisation of cases for reporting purposes, based on the available data at the national level [14]. As data collection is similar for other infectious disease, we suggest that these points are relevant to infectious disease in general.

However, TESSy has its limitations and relies—to a certain degree—upon each country’s reporting infrastructure and the timeliness of such reporting. For example, data may be collected from compulsory or voluntary collection systems, which may also be comprehensive or sentinel. Countries may also operate different detection methodologies (e.g. PCR vs culture). Further details on data collection systems and their differences are provided elsewhere (http://atlas.ecdc.europa.eu/public/index.aspx). Data quality may also vary, for example, in the recording of cases associated with foreign travel [34]. Within this study, 6.8% of cases were excluded due to foreign travel, yet travel status was unknown for 36.9% of cases. Data from enhanced surveillance studies suggest that the proportion of cases related to travel abroad may be as high as 20% [4]. Furthermore, a major limitation of TESSy data is that reported campylobacteriosis cases are only a small proportion of the overall symptomatic infections [35]. Under-ascertainment and under-reporting are likely to vary between countries, implying that small differences in incidence between countries may not be true differences [36].

However, reporting, data quality and infrastructure biases are unlikely to vary dramatically by season (i.e. it is unlikely that reporting is more complete in one month in comparison to another) [21]. Therefore, TESSy data are useful for studies exploring seasonality and the factors influencing this, as illustrated here. Of more interest from a time-series perspective is that the reporting date within TESSy is not standardised between countries. Although most countries use date of notification, others may use date of onset or date of diagnosis as the date used for statistics. Previous research indicates that the time lag between the date of notification and date of onset may be around 2 weeks for campylobacteriosis cases [11].

In this study, TESSy data were used at the national level, meaning that variations in seasonality over very large countries such as France (> 550,000 km^2^) or Sweden (> 13 degrees of latitude) were not considered. For example, across different regions of England variations in campylobacteriosis seasonality [4] and associations with weather variables have been found. Some TESSy data are available at NUTS3 level (country subdivisions into regions of 150,000–800,000 populations), but reporting at this level is incomplete for many countries. Previous studies have also explored differential impacts, such as the age of affected persons. Further studies are needed to asses these finding at the subnational level and by age group.

In spite of several European countries’ interventions to control food-borne illness, the strength and timing of campylobacteriosis peaks have remained broadly similar over the past 10 years. Nordic countries have a pronounced seasonal peak of campylobacteriosis in mid- to late summer, while most other European countries have a less pronounced peak earlier in the year. Furthermore, TESSy is a useful dataset for cross-European seasonal analysis of campylobacteriosis and other infectious diseases, but the data it contains have their limitations and rely—to a certain degree—upon each country’s reporting infrastructure and timeliness of reporting.
